# SAHA (Vorinostat) Corrects Inhibitory Synaptic Deficits Caused by Missense Epilepsy Mutations to the GABA_A_ Receptor γ2 Subunit

**DOI:** 10.3389/fnmol.2018.00089

**Published:** 2018-03-23

**Authors:** Nela Durisic, Angelo Keramidas, Christine L. Dixon, Joseph W. Lynch

**Affiliations:** Queensland Brain Institute, The University of Queensland, Brisbane, QLD, Australia

**Keywords:** epilepsy, GABA receptors, synaptic inhibition, proteostasis, febrile seizures, suberanilohydroxamic acid

## Abstract

The GABA_A_ receptor (GABA_A_R) α1 subunit A295D epilepsy mutation reduces the surface expression of α1^A295D^β2γ2 GABA_A_Rs via ER-associated protein degradation. Suberanilohydroxamic acid (SAHA, also known as Vorinostat) was recently shown to correct the misfolding of α1^A295D^ subunits and thereby enhance the functional surface expression of α1^A295D^β2γ2 GABA_A_Rs. Here we investigated whether SAHA can also restore the surface expression of γ2 GABA_A_R subunits that incorporate epilepsy mutations (N40S, R43Q, P44S, R138G) known to reduce surface expression via ER-associated protein degradation. As a control, we also investigated the γ2*^K289M^* epilepsy mutation that impairs gating without reducing surface expression. Effects of mutations were evaluated on inhibitory postsynaptic currents (IPSCs) mediated by the major synaptic α1β2γ2 GABA_A_R isoform. Recordings were performed in neuron-HEK293 cell artificial synapses to minimise contamination by GABA_A_Rs of undefined subunit composition. Transfection with α1β2γ2*^N40S^*, α1β2γ2*^R43Q^*, α1β2γ2*^P44S^* and α1β2γ2*^R138G^* subunits produced IPSCs with decay times slower than those of unmutated α1β2γ2 GABA_A_Rs due to the low expression of mutant γ2 subunits and the correspondingly high expression of slow-decaying α1β2 GABA_A_Rs. SAHA pre-treatment significantly accelerated the decay time constants of IPSCs consistent with the upregulation of mutant γ2 subunit expression. This increase in surface expression was confirmed by immunohistochemistry. SAHA had no effect on either the IPSC kinetics or surface expression levels of α1β2γ2*^K289M^* GABA_A_Rs, confirming its specificity for ER-retained mutant γ2 subunits. We also found that α1β2γ2*^K289M^* GABA_A_Rs and SAHA-treated α1β2γ2*^R43Q^*, α1β2γ2*^P44S^* and α1β2γ2*^R138G^* GABA_A_Rs all mediated IPSCs that decayed at significantly faster rates than wild type receptors as temperature was increased from 22 to 40°C. This may help explain why these mutations cause febrile seizures (FS). Given that SAHA is approved by therapeutic regulatory agencies for human use, we propose that it may be worth investigating as a treatment for epilepsies caused by the N40S, R43Q, P44S and R138G mutations. Although SAHA has already been proposed as a therapeutic for patients harbouring the α1^A295D^ epilepsy mutation, the present study extends its potential utility to a new subunit and four new mutations.

## Introduction

The epilepsies are a related group of neurological disorders characterised by seizures resulting from abnormal, hypersynchronised electrical activity in neurons. Around one third of epilepsy patients have seizures that are refractory to current pharmacotherapy (Tang et al., 2017). Both genetic and environmental factors contribute to the pathological mechanisms underlying the epilepsies and it is generally considered that an improved understanding of the molecular and cellular mechanisms of epileptogenesis may reveal novel therapeutic opportunities.

Many hereditary epilepsy mutations are found in the GABA type-A receptors (GABA_A_Rs) that are responsible for mediating most of the synaptic and extrasynaptic inhibition in the brain. Mutations to GABA_A_R α1, β3, γ2 and δ subunits are associated with a wide variety of epilepsy syndromes ranging from relatively benign childhood absence epilepsies to exceptionally severe forms such as Dravet’s syndrome (Macdonald et al., 2010; Hirose, 2014; Kang and Macdonald, 2016). These mutations are invariably loss-of-function, with the severity of the symptoms frequently correlating with the severity of the impairment to GABA_A_R function or surface expression (Kang and Macdonald, 2016). Drugs that potentiate GABA_A_Rs, such as benzodiazepines, neurosteroids and barbiturates, are often effective in treating epilepsies whereas drugs that inhibit GABA_A_Rs, such as bicuculline and picrotoxin, can give rise to seizures (Rogawski and Löscher, 2004; Riss et al., 2008). Together, this is consistent with the widely held view that GABAergic inhibition restrains the tendency of recurrently connected excitatory neural networks to transition, via positive feedback, into synchronous epileptiform activity (Rogawski and Löscher, 2004).

GABA_A_Rs belong to the family of pentameric ligand-gated ion channels and are constructed from a family of 19 subunits (α1–6, β1–3, γ1–3, δ, ε, π, θ and ρ1–3). The majority of GABA_A_Rs found *in vivo* incorporate two α subunits, two β subunits and a single γ or δ subunit, with the most abundant synaptic subtype comprising α1, β2 and γ2 subunits in an α1-β2-α1-γ2-β2 stoichiometry (Olsen and Sieghart, 2009). The γ2 subunit, which is widely expressed throughout the brain, is essential for clustering GABA_A_Rs at the synapse (Thomson and Jovanovic, 2010).

The γ2 subunit is a frequent target of GABA_A_R epilepsy mutations (Kang and Macdonald, 2016) with the following five γ2 missense mutations having been characterised in detail: N40S, R43Q, P44S, R138G and K289M. This numbering omits the signal peptide. If signal peptide is included, residue numbering would be: N79S, R82Q, P83S, R177G and K328M. The K289M mutation has been shown to impair inhibitory synaptic signalling by accelerating the decay rate of GABAergic inhibitory postsynaptic currents (IPSCs; Bianchi et al., 2002; Eugene et al., 2007) whereas the other four mutations all reduce the surface expression of functional GABA_A_Rs to varying degrees by enhancing the rate of endoplasmic reticulum (ER)-associated protein degradation (Sancar and Czajkowski, 2004; Hales et al., 2005; Kang et al., 2006; Tan et al., 2007; Chaumont et al., 2013; Huang et al., 2014; Todd et al., 2014).

The α1 subunit A295D epilepsy mutation also enhances the rate of ER-associated protein degradation (Gallagher et al., 2005, 2007). This has the effect of almost completely ablating the surface expression of α1^A295D^β2γ2 GABA_A_Rs. However, the surface expression of functional α1^A295D^β2γ2 GABA_A_Rs was partially restored by exposure to a 2.5 μM concentration of the proteostatic enhancer, suberanilohydroxamic acid (SAHA, also known as Vorinostat; Di et al., 2013). We recently confirmed this by demonstrating in an artificial GABAergic synapse preparation that although α1^A295D^β2γ2 GABA_A_Rs were normally unable to mediate IPSCs, pre-exposure to 0.1 μM SAHA induced α1^A295D^β2γ2 GABA_A_Rs to mediate robust IPSCs that were indistinguishable in magnitude and waveform to those mediated by unmutated α1β2γ2 receptors (Chen et al., 2017a).

SAHA is approved by drug regulatory authorities worldwide for the management of cutaneous T-cell lymphoma and is currently being investigated for other indications including prostate cancer, leukemia, breast cancer, glioma and lung cancer (Iwamoto et al., 2013; Bubna, 2015). Because SAHA crosses the blood-brain barrier and is evidently safe for human internal use, the above findings suggest it may be worthy of investigation as a treatment for epilepsies caused by the α1^A295D^ mutation. In the present study we extended this line of investigation by asking whether SAHA can recover the surface expression of epilepsy mutations to γ2 subunits that also result in ER-retention.

It is difficult to study a specific GABA_A_R isoform in native neuronal synapses due to the large number of possible GABA_A_R isoforms that may be expressed in any neuron type. Although recombinantly expressing GABA_A_Rs in a heterologous expression system (e.g., HEK293 cells) allows individual isoforms to be studied in isolation, GABA must be artificially applied and thus it cannot reliably mimic the dynamic GABA concentration profile that exists in a synapse. Both problems can be solved simultaneously via the generation of “artificial synapses” between neurons and HEK293 cells that express the GABA_A_R isoform of interest (Dong et al., 2007; Brown et al., 2014; Dixon et al., 2015b). By generating GABAergic synapses that incorporate defined subunit combinations, it is possible to determine how a given epilepsy-causing GABA_A_R mutation disrupts synaptic function. In this study we aimed to investigate: (1) the extent to which GABAergic inhibitory signalling is impaired by the γ2 subunit N40S, R43Q, P44S, R138G or K289M mutations; and (2) whether SAHA can recover these deficits.

## Materials and Methods

### Cell Culture, Transfection and Artificial Synapse Formation

Methods for preparing neurons and HEK293 cells for artificial synapse recordings have previously been detailed (Dixon et al., 2015b). Briefly, HEK293 cells were transfected with cDNAs encoding human α1, β2 and γ2L GABA_A_R subunits (all in the pcDNA3.1 plasmid) and co-transfected with pEGFP and neuroligin 2A (in pNICE) at a ratio of 1:1:4:1:1, using a calcium-phosphate co-precipitation protocol. Mutations to the γ2 subunit were introduced by site-directed mutagenesis and confirmed by DNA sequencing of the entire plasmid. We have previously shown that the 1:1:4 transfection protocol results in the expression of >90% of triheteromeric α1β2γ2 GABA_A_Rs and <10% diheteromeric α1β2 GABA_A_Rs (Dixon et al., 2014). Euthanasia of timed-pregnant rats was performed via CO_2_ inhalation as approved by the University of Queensland Animal Ethics Committee (approval number: QBI/142/16/NHMRC/ARC). The cortices of e18 rat embryos were dissected out, triturated and plated on poly-D-lysine coated coverslips at a density of ~80 × 10^3^ cells per coverslip. The cells were plated into Dulbecco’s modified Eagles medium with 10% fetal bovine serum and this was replaced after 24 h with Neurobasal medium, including 2% B27 and 1% glutamax. After 1 week, half of this medium was replaced with fresh medium. Neurons were allowed to grow for 3–4 weeks before freshly transfected HEK293 cells were plated onto the neurons. Artificial synaptic connections typically formed within 24 h and IPSCs in HEK293 cells were recorded by whole-cell patch clamp between 2–5 days later. In experiments involving the pre-application of SAHA, we added 2.5 μM SAHA to the co-cultures at the time of HEK293 cell plating and waited 3 days before recording.

### Electrophysiology

All artificial synapse recordings were performed via whole-cell patch clamp recording at a holding potential of −70 mV. The intracellular solution was composed of (in mM): 145 CsCl, 2 CaCl_2_, 2 MgCl_2_, 10 HEPES, and 10 EGTA, adjusted to pH 7.4 with CsOH. Cells were perfused with extracellular solution, which contained (in mM): 140 NaCl, 5 KCl, 2 CaCl_2_, 1 MgCl_2_, 10 HEPES, and 10 D-glucose, adjusted to pH 7.4 with NaOH. Currents were filtered (−3 dB, 4-pole Bessel) at 4 kHz and sampled at 10 kHz and recorded using a Multiclamp 700B amplifier and pClamp 10 software (Molecular Devices, Sunnyvale, CA, USA). Recordings with series resistances >20 MΩ were discarded and series resistance compensation was not applied to the recorded cell. The temperature of the bath was increased from room temperature 22 ± 2°C to 40 ± 1°C using an in-line bath heater (Warner TC-324B, Hamden, CT, USA).

Single channel and macropatch recordings were made from excised outside-out patches from HEK293 cells expressing either wild-type α1β2γ2 or α1β2γ2*^K289M^* receptors, using an Axon 200B amplifier and pClamp 10 software (Molecular Devices, Sunnyvale, CA, USA). Unless otherwise indicated, currents were recorded at a clamped potential of −70 mV, low-pass filtered at 5 kHz and digitised at 20 kHz. Rapid solution exchange was achieved by lateral switching of the solution flowing over the recorded macropatch using a piezoelectric translator (Siskiyou, Grants Pass, OR, USA). Single channel and macropatch currents were analysed using pClamp 10, SigmaPlot 13 and QuB software. The statistical significance threshold was set at *p* < 0.01 for single channel and macropatch experiments.

### Immunohistochemistry

To determine the cell surface expression levels of GABA_A_Rs, live HEK293 cells were incubated for 1 h with primary antibodies directed against surface epitopes of γ2 subunits (1:100, rabbit, #GA-005, Alomone Labs, Jerusalem, Israel) in equilibrated DMEM containing 1% BSA at 37°C. Cells were then washed and fixed with 4% paraformaldehyde in phosphate buffered saline (PBS) for 10 min. After washing with PBS, cells were blocked for 1 h with 1% bovine serum albumen (BSA) in PBS and then incubated with the secondary donkey anti-rabbit antibodies (1:50, Jackson ImmunoResearch, West Grove, PA, USA) in PBS with 1% BSA for 1 h at room temperature. The secondary antibodies were labelled in-house with Cy3 dye.

### Microscopy

All imaging experiments were carried out with a commercial NSTORM microscope (Nikon Instruments, Japan). Laser light at 560 nm was used to excite Cy3. The emitted light was collected by an oil immersion 100×, 1.49 NA objective, filtered by an emission filter (BP 605/52), and imaged onto an EMCCD camera (Andor 897 EMCCD, UK) at an exposure time of 50 ms per frame. Images were analysed in Fiji software (NIH).

### Statistical Analyses

Data sets were first tested for normal distribution prior to using analysis of variance (ANOVA) tests and Tukey’s *post hoc* tests to determine statistical significance. In all experimental analyses, * and ** represent significance levels of *p* < 0.05 and *p* < 0.01 respectively. The tests were conducted with SigmaPlot software. All data are presented as mean ± SEM.

## Results

### Properties of IPSCs Generated by GABA_A_Rs Incorporating Epilepsy-Causing γ2 Mutant Subunits

**Figure 1:**
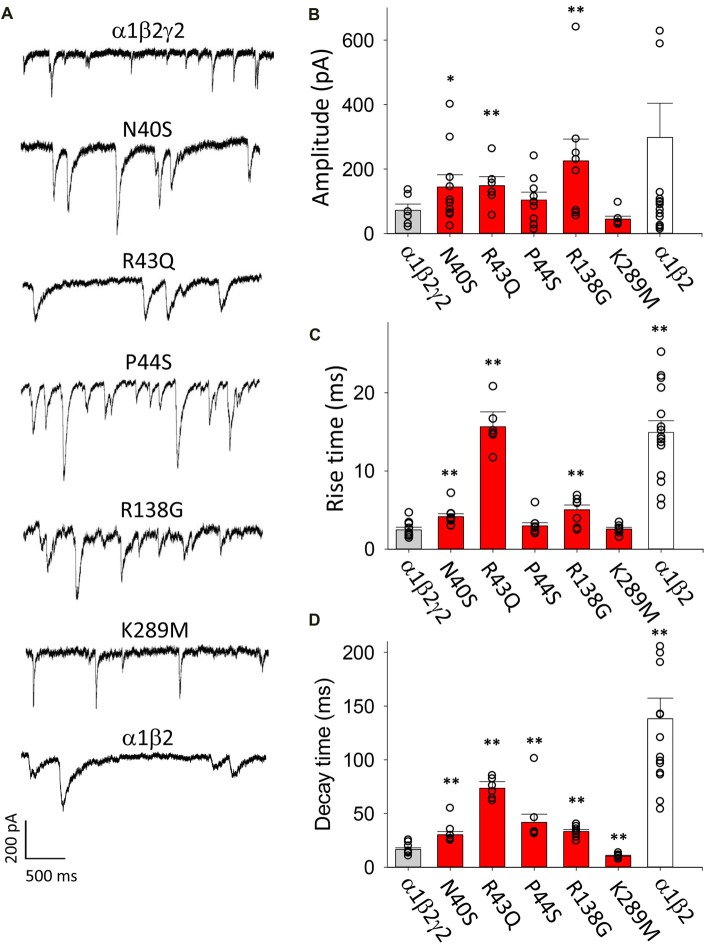
Overview of the effects of mutations on inhibitory postsynaptic current (IPSC) parameters. In this and all subsequent figures, individual data points represent the average of all well-isolated events recorded from a single cell or patch. **(A)** Sample recordings of spontaneous IPSCs recorded from HEK293 cells transfected with the indicted wild type and mutant GABA_A_ receptor (GABA_A_R) subunits. **(B)** Mean amplitude of IPSCs. Amplitudes typically varied over a wide range, and GABA_A_Rs with N40S, R43Q and R138G exhibited a significantly larger amplitude than α1β2γ2 GABA_A_Rs. **(C)** Mean IPSC 10%–90% rise times. Most ER-retained mutant α1β2γ2 GABA_A_Rs exhibited significantly slower rise times than α1β2γ2 GABA_A_Rs. **(D)** Mean IPSC decay time constants. All ER-retained mutant α1β2γ2 GABA_A_Rs exhibited significantly slower decay time constants than α1β2γ2 GABA_A_Rs, whereas the α1β2γ2*^K289M^* GABA_A_Rs exhibited a significantly faster decay time constant than α1β2γ2 GABA_A_Rs. *p < 0.05 and **p < 0.01.

A sample voltage-clamp recording of spontaneous IPSCs mediated by α1β2γ2 GABA_A_Rs in artificial synapses is shown in Figure 1A (top panel). We collected all well-isolated IPSCs from each cell and averaged their amplitudes to generate a single data point. The mean amplitudes of IPSCs recorded from cells transfected with α1, β2 and γ2 subunits are shown in Figure 1B (gray bar). We normalised and digitally averaged the IPSCs from each individual cell, and from the resulting waveform we measured a single mean 10%–90% rise time and a decay time constant value for each cell. These data, pooled from single digitally averaged IPSCs obtained from each cell, are presented in Figures 1C,D (gray bars).

As a negative control for γ2 subunit incorporation, we repeated these experiments on IPSCs mediated by α1β2 GABA_A_Rs. Figure 1A (bottom panel) displays sample recordings of IPSCs in artificial synapses that incorporated α1β2 GABA_A_Rs only. As shown in Figure 1B (white bar), the mean IPSC amplitudes varied widely from cell to cell and were not significantly different from α1β2γ2 GABA_A_Rs. However, the 10%–90% activation times and decay time constants of IPSCs mediated by α1β2 GABA_A_Rs were significantly slower than those mediated by α1β2γ2 GABA_A_Rs (Figures 1C,D, white bars). The slow decay time constant is expected given that α1β2 GABA_A_Rs deactivate more slowly than α1β2γ2 GABA_A_Rs (Bowser et al., 2002). The slower rise times suggest the diheteromeric receptors may be localised at extrasynaptic or perisynaptic sites (Wu et al., 2012).

Next we transfected HEK293 cells with α1, β2 and N40S, R43Q, P44S R138G or K289M mutant γ2 subunits and analyzed IPSCs mediated by the assembled GABA_A_Rs. The amplitudes of IPSCs mediated by cells transfected with γ2*^N40S^*, γ2*^R43Q^* and γ2*^R138G^* subunits were significantly larger than those mediated by α1β2γ2 GABA_A_Rs, whereas the other mutations did not affect this parameter (Figure 1B, red bars). The IPSC rise times and decay time constants were also differentially affected by the mutations (Figures 1C,D, red bars). All mutants, with the exception of K289M and P44S, mediated IPSCs with rise times that were significantly slower than those of α1β2γ2 GABA_A_Rs. The decay time constants of the currents mediated by GABA_A_Rs incorporating all mutations except K289M were longer than those of α1β2γ2 receptors. Consistent with previous findings (Bianchi et al., 2002; Eugene et al., 2007), α1β2γ2*^K289M^* GABA_A_Rs caused a significant acceleration of the IPSC decay time constant relative to α1β2γ2 GABA_A_Rs (Figure 1D).

Since the N40S, R43Q, P44S and R138G mutations all cause ER retention of γ2 subunits, it is possible that the slower rise and decay times of IPSCs mediated by GABA_A_Rs incorporating these mutations could be due to a reduction in expression of γ2-containing triheteromeric receptors and a corresponding increase in the expression of α1β2 diheteromeric GABA_A_Rs. However, it is also possible that the mutations modulated the intrinsic gating properties of triheteromeric GABA_A_Rs.

### Effect of SAHA on the Cell Surface Expression Levels of Wild Type and Mutant GABA_A_Rs

**Figure 2:**
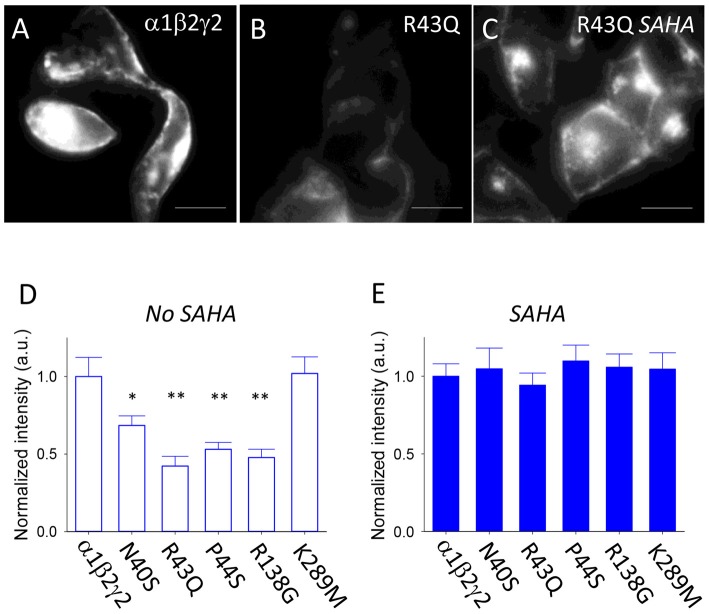
Effect of suberanilohydroxamic acid (SAHA) on the surface expression of wild type and mutant GABA_A_Rs. **(A)** Sample image of HEK293 cells expressing α1β2γ2 GABA_A_Rs. The scale bar represents 10 μm. **(B)** Sample image of HEK293 cells expressing α1β2γ2*^R43Q^* GABA_A_Rs. **(C)** Sample image of HEK293 cells expressing α1β2γ2*^R43Q^* GABA_A_Rs following SAHA pre-application. **(D)** In the absence of SAHA pre-application, all mutants except γ2*^K298M^* showed reduced surface expression relative to wild type (20–60 cells per mutant). **(E)** After SAHA pre-application, the surface expression levels of all mutants were not significantly different to wild type. *p < 0.05 and **p < 0.01.

SAHA is a histone deacetylase inhibitor that corrects protein folding and improves the assembly and surface expression of triheteromeric GABA_A_Rs that incorporate the ER-retained A295D epilepsy mutant α1 subunit (Di et al., 2013; Chen et al., 2017a). To evaluate the extent to which SAHA improves the assembly, trafficking and surface expression of GABA_A_Rs incorporating mutant γ2 subunits, we used primary antibodies that recognise an extracellular epitope of the γ2 subunit as detailed in “Materials and Methods” section. These antibodies are suitable for live cell imaging. Transfected HEK293 cells were analyzed in isolation (i.e., not in artificial synapses with neurons). Live HEK293 cells were then incubated with primary antibodies, fixed and then Cy3-labelled secondary antibodies were applied. This enabled us to specifically label GABA_A_Rs expressed at the plasma membrane. Figure 2A shows an example of HEK293 cells transfected with α1, β2 and γ2 subunits. In contrast, HEK293 cells transfected with α1, β2 and γ2*^R43Q^* subunits exhibited reduced fluorescence consistent with low surface expression levels (Figure 2B). SAHA pre-application (2.5 μM for 3 days) resulted in a dramatic increase in fluorescence suggesting enhanced surface expression of γ2*^R43Q^*-containing receptors (Figure 2C). We performed these experiments on wild type and all five mutant receptors. As expected, in the absence of SAHA pre-application, all mutants except γ2*^K298M^* showed reduced surface expression relative to wild type (Figure 2D, 20–60 cells per mutant). After pre-incubation with SAHA, the surface expression levels of all mutants were not significantly different to wild type (Figure 2E). Together these data suggest that SAHA enhances the surface expression of all GABA_A_Rs with epilepsy-causing mutations that result in ER retention.

### Effects of Mutations and SAHA on GABAergic IPSCs in Artificial Synapses

We next sought to investigate the effects of SAHA on the kinetic properties of IPSCs mediated by GABA_A_Rs incorporating mutant γ2 subunits. Since all mutants except N40S are associated with febrile seizures (FS), we recorded IPSCs at room temperature (22°C) and at 40°C following incubation with SAHA.

#### Transfection With α1 and β2

**Figure 3:**
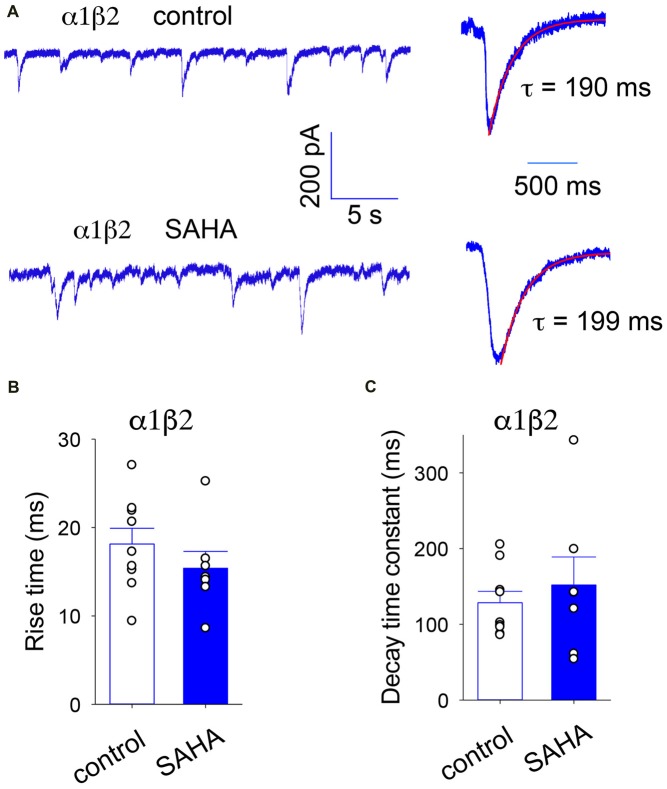
IPSCs mediated by α1β2 GABA_A_Rs are not affected by SAHA. **(A)** Sample recordings of spontaneous IPSCs recorded from HEK293 cells expressing α1β2 GABA_A_Rs without SAHA pre-application (upper trace) and following a 3 day application of 0.1 μM SAHA (lower trace). The right hand panels show digitally averaged and normalised IPSCs from the recordings as displayed on the left. The results of single exponential fits to IPSC decay phases are shown. **(B)** Mean IPSC 10%–90% rise times. **(C)** Mean IPSC decay time constants. SAHA had no significant effect on either parameter.

Our first experiment was to investigate the effect of SAHA pretreatment on α1β2 diheteromeric GABA_A_Rs as a control. Sample recordings of IPSCs mediated α1β2 GABA_A_Rs recorded with or without SAHA pretreatment are shown in Figure 3A, together with digitally averaged, normalised IPSCs from the same recordings. The digitally averaged traces suggest no significant effect of SAHA. Indeed, mean IPSC rise times (Figure 3B) and decay time constants (Figure 3C) revealed no significant effect of SAHA.

#### Transfection With α1, β2 and γ2*^N40S^*

**Figure 4:**
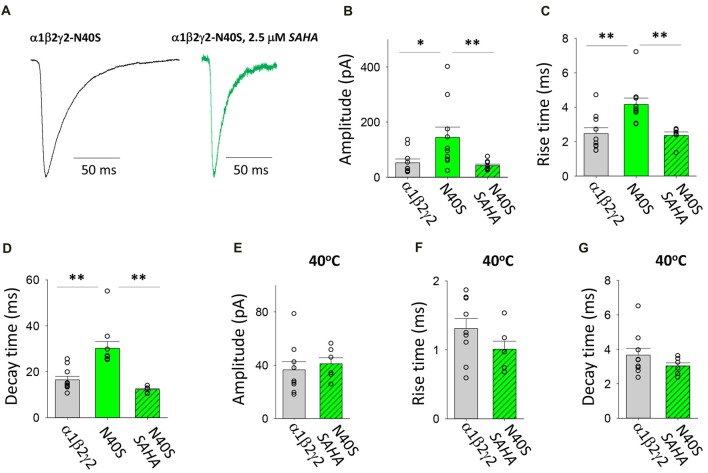
Effects of SAHA and temperature on IPSCs mediated by α1β2γ2*^N40S^* GABA_A_Rs. **(A)** Examples of digitally averaged and normalised IPSCs from HEK293 cells transfected with α1, β2 and γ2*^N40S^* subunits with and without SAHA pre-application. **(B–D)** Effect of SAHA pre-application on the amplitudes, 10%–90% rise times and decay time constants of IPSCs mediated by α1β2γ2*^N40S^* GABA_A_Rs. The control data for α1β2γ2 and α1β2γ2*^N40S^* GABA_A_Rs have been replotted from Figure 1. All data in panels **(A–D)** were recorded at room temperature (22°C). **(E–G)** Mean amplitudes, 10%–90% rise times and decay time constants of IPSCs mediated by α1β2γ2 and α1β2γ2*^N40S^* GABA_A_Rs at 40°C. *p < 0.05 and **p < 0.01.

N40S is a heterozygous mutation originally found in a patient with generalised tonic-clonic seizures (GTCS) without FS (Shi et al., 2010). It was shown to alter the steepness of the GABA dose-response relationship and to cause a modest (12%) reduction in γ2 surface expression (Migita et al., 2013; Huang et al., 2014). Examples of digitally averaged and normalised IPSCs mediated by GABA_A_Rs with and without pre-incubation with SAHA are shown in Figure 4A. At room temperature (22°C), the assembled GABA_A_Rs produced IPSCs with significantly larger amplitudes, rise times and decay time constants compared to those produced by α1β2γ2 GABA_A_Rs (Figures 4B–D). All of these effects were completely reversed by SAHA. Thus, following SAHA preincubation, IPSCs mediated by α1β2γ2*^N40S^* GABA_A_Rs became indistinguishable from those mediated by α1β2γ2 GABA_A_Rs. Although the kinetic parameters of IPSCs were dramatically accelerated at 40°C, we did not observe a statistically significant difference in amplitude, rise or decay times between α1β2γ2 and α1β2γ2*^N40S^* GABA_A_Rs (Figures 4E–G). Our results are consistent with SAHA enhancing the surface expression of α1β2γ2*^N40S^* GABA_A_Rs to levels similar to those of α1β2γ2 receptors. Finally, the previously reported absence of an FS phenotype is consistent with the absence of a differential effect of elevated temperature on IPSC properties.

#### Transfection With α1, β2 and γ2*^R43Q^*

**Figure 5:**
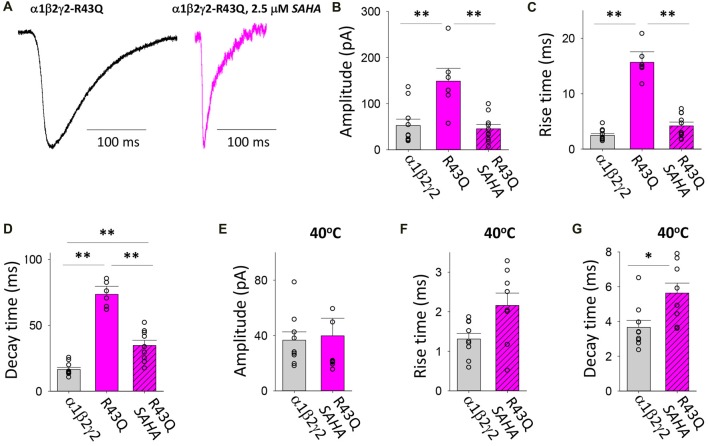
Effects of SAHA and temperature on IPSCs mediated by α1β2γ2*^R43Q^* GABA_A_Rs. **(A)** Examples of digitally averaged and normalised IPSCs from HEK293 cells transfected with α1, β2 and γ2*^R43Q^* subunits with and without SAHA pre-application. **(B–D)** Effect of SAHA pre-application on the amplitudes, 10%–90% rise times and decay time constants of IPSCs mediated by α1β2γ2*^R43Q^* GABA_A_Rs. The control data for α1β2γ2 and α1β2γ2*^R43Q^* GABA_A_Rs have been replotted from Figure 1. All data in panels **(A–D)** were recorded at room temperature (22°C). **(E–G)** Mean amplitudes, 10%–90% rise times and decay time constants of IPSCs mediated by α1β2γ2 and α1β2γ2*^R43Q^* GABA_A_Rs at 40°C. *p < 0.05 and **p < 0.01.

The R43Q mutation is associated with a heterozygous form of childhood absence epilepsy (CAE) with FS (Wallace et al., 2001). The mutation prevents the oligomerisation of γ2 and β2 subunits (Hales et al., 2005) which results in a dramatic reduction in the surface expression of assembled receptors and the ER retention of mutant γ2 subunits (Kang and Macdonald, 2004; Sancar and Czajkowski, 2004; Eugene et al., 2007; Frugier et al., 2007). When expressed in triheteromeric GABA_A_Rs, the γ2*^R43Q^* mutation slows deactivation due to slowed GABA unbinding and slowed recovery from desensitisation (Goldschen-Ohm et al., 2010). We found that transfection with γ2*^R43Q^* mutant significantly increased the magnitude and significantly slowed both the rise and decay times of IPSCs (Figures 5A–D). The mean IPSC amplitudes and rise times mediated by assembled GABA_A_Rs were both significantly decreased following SAHA pre-application to became comparable to those mediated by α1β2γ2 GABA_A_Rs (Figures 5A–C). The mean IPSC decay time constant also decreased significantly, however it remained significantly slower than that of α1β2γ2 GABA_A_Rs (Figures 5A,D). Increasing the temperature to 40°C resulted in similar reductions in IPSC amplitude for α1β2γ2 and α1β2γ2*^R43Q^* GABA_A_Rs (Figure 5E). Although not statistically different when compared to α1β2γ2 GABA_A_Rs, a trend towards slower IPSC rise times was observed in α1β2γ2*^R43Q^* GABA_A_Rs at 40°C (Figure 5F). However, at 40°C the IPSC decay time constant remained significantly slower than that of α1β2γ2 GABA_A_Rs. Together these findings indicate that while substantial, recovery of α1β2γ2*^R43Q^* surface expression by SAHA may be incomplete and that the slow decay times of IPSCs may be due to a significant population of α1β2 GABA_A_Rs being trafficked to the plasma membrane. Alternately, the slow IPSC decay time may be due to the slower channel deactivation of α1β2γ2*^R43Q^* GABA_A_Rs (Goldschen-Ohm et al., 2010).

#### Transfection With α1, β2 and γ2*^P44S^*

**Figure 6:**
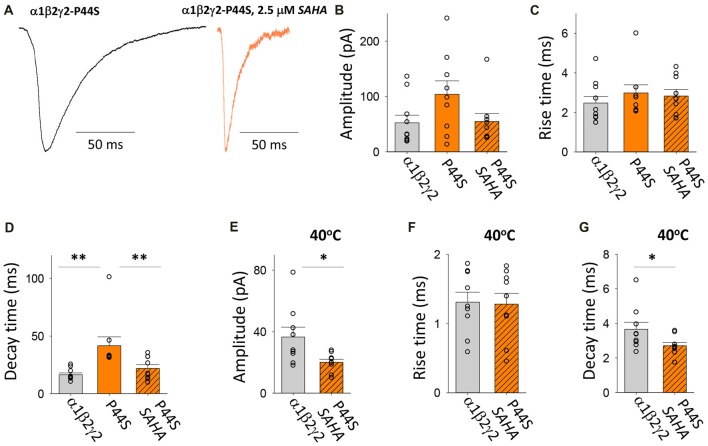
Effects of SAHA and temperature on IPSCs mediated by α1β2γ2*^P44S^* GABA_A_Rs. **(A)** Examples of digitally averaged and normalised IPSCs from HEK293 cells transfected with α1, β2 and γ2*^P44S^* subunits with and without SAHA pre-application. **(B–D)** Effect of SAHA pre-application on the amplitudes, 10%–90% rise times and decay time constants of IPSCs mediated by α1β2γ2*^P44S^* GABA_A_Rs. The control data for α1β2γ2 and α1β2γ2*^P44S^* GABA_A_Rs have been replotted from Figure 1. All results in panels **(A–D)** were recorded at room temperature (22°C). **(E–G)** Mean amplitudes, 10%–90% rise times and decay time constants of IPSCs mediated by α1β2γ2 and α1β2γ2*^P44S^* GABA_A_Rs at 40°C. *p < 0.05 and **p < 0.01.

This autosomal dominant (AD) mutation was identified in families with genetic epilepsy with febrile seizures plus (GEFS+; Lachance-Touchette et al., 2011). As with the R43Q mutation, it causes a dramatic reduction in the surface expression of assembled receptors and the ER retention of mutant γ2 subunits (Huang et al., 2014). Consistent with this, our measurements indicate that spontaneous IPSCs recorded with and without SAHA pre-application have different properties (Figure 6A). A trend toward lower IPSC amplitudes was observed with SAHA pre-application, but this failed to reach statistical significance (Figure 6B). Analysis of IPSC kinetics showed that GABA_A_Rs assembled after transfection with α1, β2 and γ2*^P44S^* subunits mediated IPSCs with similar rise times to α1β2γ2 GABA_A_Rs and that SAHA application did not significantly change this parameter (Figure 6C). However, the transfection with γ2*^P44S^* caused IPSCs mediated by GABA_A_Rs to decay significantly more slowly than when γ2 was transfected instead (Figure 6D). This effect was completely reversed by SAHA pre-application (Figure 6D). Increasing the temperature from 22 to 40°C reduced the amplitude of IPSCs mediated by α1β2γ2*^P44S^* GABA_A_Rs to a significantly greater extent than those mediated by α1β2γ2 GABA_A_Rs (Figure 6E). While IPSC rise times were not differentially affected by increasing the temperature (Figure 6F), the IPSC decay time constant was reduced significantly more than that of α1β2γ2 GABA_A_Rs (Figure 6G). Taken together these data suggest that surface expression of α1β2γ2*^P44S^* GABA_A_Rs is restored to wild-type receptor levels by SAHA and that they are also more susceptible than α1β2γ2 GABA_A_Rs to the effects of elevated temperature.

#### Transfection With α1, β2 and γ2*^R138G^*

**Figure 7:**
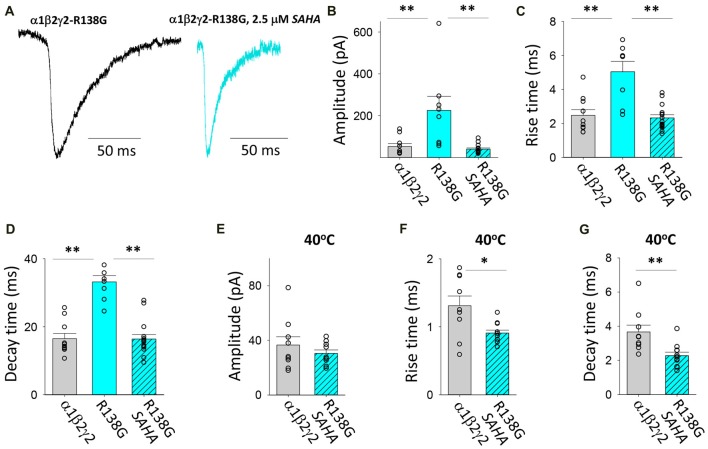
Effects of SAHA and temperature on IPSCs mediated by α1β2γ2*^R138G^* GABA_A_Rs. **(A)** Examples of digitally averaged and normalised IPSCs from HEK293 cells transfected with α1, β2 and γ2*^R138G^* subunits s with and without SAHA pre-application. **(B–D)** Effect of SAHA pre-application on the amplitudes, 10%–90% rise times and decay time constants of IPSCs mediated by α1β2γ2*^R138G^* GABA_A_Rs. The control data for α1β2γ2 and α1β2γ2*^R138G^* GABA_A_Rs have been replotted from Figure 1. All results in panels **(A–D)** were recorded at room temperature (22°C). **(E–G)** Mean amplitudes, 10% –90% rise times and decay time constants of IPSCs mediated by α1β2γ2 and α1β2γ2*^R138G^* GABA_A_Rs at 40°C. *p < 0.05 and **p < 0.01.

This AD mutation was identified in a family with complex FS where it was originally reported to enhance the triheteromeric receptor desensitisation rate (Audenaert et al., 2006). R138G was subsequently shown to decrease the surface expression of assembled triheteromeric receptors and induce mutant γ2 subunits to be retained in the ER (Todd et al., 2014). Evidence was also presented that the γ2*^R138G^* subunit altered GABA_A_R composition by allowing a β2 subunit to take the place of the mutant γ2 subunit (Todd et al., 2014). We found that the GABA_A_Rs formed after transfection with α1, β2 and γ2*^R138G^* subunits mediated IPSCs with significantly enhanced IPSC magnitudes and slower IPSC rise and decay times relative to those mediated by α1β2γ2 receptors (Figures 7A–D). Moreover, SAHA pre-treatment resulted in each of the three parameters reverting to unmutated receptor values (Figures 7A–D), suggesting a dramatic enhancement in GABA_A_R assembly and trafficking. At 40°C, α1β2γ2*^R138G^* and α1β2γ2 GABA_A_Rs mediated IPSCs with similar amplitudes (Figure 7E). However, the increase in temperature from 22 to 40°C caused IPSCs mediated by α1β2γ2*^R138G^* GABA_A_Rs to activate and deactivate more rapidly than those mediated by α1β2γ2 GABA_A_Rs. Thus, our results show that α1β2γ2*^R138G^* GABA_A_Rs have an altered temperature sensitivity which could underlie susceptibility to FS.

#### Transfection With α1, β2 and γ2*^K289M^*

**Figure 8:**
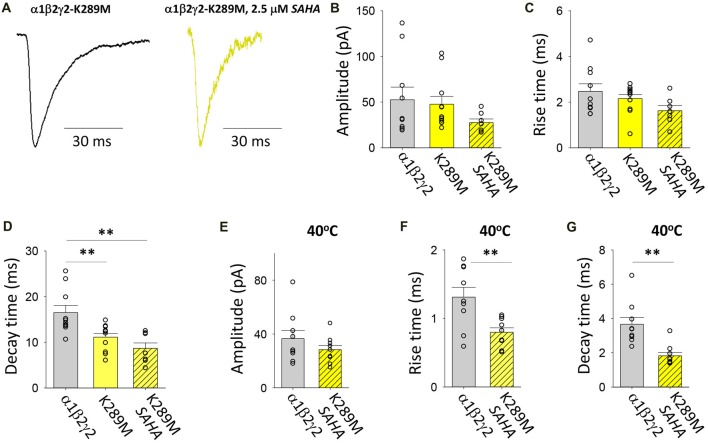
Effects of SAHA and temperature on IPSCs mediated by α1β2γ2*^K289M^* GABA_A_Rs. **(A)** Examples of digitally averaged and normalised IPSCs from HEK293 cells transfected with α1, β2 and γ2*^K289M^* subunits with and without SAHA pre-application. **(B–D)** Effect of SAHA pre-application on the amplitudes, 10%–90% rise times and decay time constants of IPSCs mediated by α1β2γ2*^K289M^* GABA_A_Rs. The control data for α1β2γ2 and α1β2γ2*^K289M^* GABA_A_Rs have been replotted from Figure 1. All results in panels **(A–D)** were recorded at room temperature (22°C). **(E–G)** Mean amplitudes, 10%–90% rise times and decay time constants of IPSCs mediated by α1β2γ2 and α1β2γ2*^K289M^* GABA_A_Rs at 40°C. **p < 0.01.

As with P44S, this AD mutation was discovered in probands with familial GEFS+ (Baulac et al., 2001). The mutation was previously found not to impair membrane trafficking (Eugene et al., 2007; Bouthour et al., 2012) but rather to accelerate the rate of receptor deactivation (Bianchi et al., 2002) thereby reducing the decay time constant of neuronal GABAergic IPSCs (Eugene et al., 2007; Bouthour et al., 2012). Furthermore, at elevated temperatures, both the number of postsynaptic receptor clusters and the frequency of miniature IPSCs were reduced in hippocampal neurons transfected with γ2*^K289M^* subunits (Bouthour et al., 2012). Examples of digitally averaged and normalised IPSCs mediated by GABA_A_Rs assembled after transfection with α1, β2 and γ2*^K289M^* with and without SAHA pre-incubation suggest little if any effect of SAHA (Figure 8A). Indeed, at 22°C the average amplitudes of IPSCs mediated by α1β2γ2*^K289M^* and α1β2γ2 GABA_A_Rs were similar and were not significantly affected by SAHA (Figure 8B). The rise times of IPSCs mediated by α1β2γ2*^K289M^* GABA_A_Rs were also indistinguishable from those mediated by α1β2γ2 GABA_A_Rs (Figure 8C). As expected, however, the mutant IPSC decay time constant was significantly accelerated (Figure 8D). Incubation with SAHA did not affect IPSC rise or decay times, consistent with SAHA having no effect on the surface expression of α1β2γ2*^K289M^* GABA_A_Rs. At 40°C, the rise times and decay time constants of IPSCs mediated by α1β2γ2*^K289M^* GABA_A_Rs were both significantly faster than those mediated by α1β2γ2 GABA_A_Rs (Figures 8F,G) although IPSC amplitudes were little affected (Figure 8E). However, it is important to note that the decay time constant difference was not temperature-dependent since it also occurred at 22°C. The results so far suggest that the main pathomechanism for γ2*^K289M^* is not subunit misfolding or impaired trafficking, as is the case for the other γ2 mutations, but rather altered intrinsic gating of the receptor. These effects will be quantified below.

### Effects of Mutations on the Temperature Sensitivity of IPSC Rise and Decay Rates

**Figure 9:**
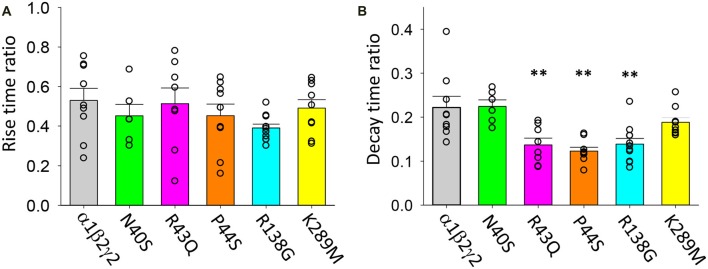
Temperature sensitivity of the rise and decay rates of IPSCs mediated by wild type and mutant GABA_A_Rs. All data points represent the ratio of digitally averaged IPSCs recorded at both 22 and 40°C. **(A)** Ratio of IPSC rise times at 40°C relative to 22°C for the indicated receptors. On average, rise times were reduced by a factor of 50% as temperature was increased and were not mutation-dependent. **(B)** Ratio of IPSC decay time constants at 40°C relative to 22°C for the indicated receptors. Receptors incorporating R43Q, P44S and R138G mutations exhibited a significantly heightened temperature sensitivity relative to unmutated receptors. **p < 0.01.

Data presented in Figures 5–7 above suggest that the mutations associated with FS (i.e., R43Q, P44S, R138G and K289M) may enhance the acceleration of the IPSC decay rate as temperature is increased from 22 to 40°C. We sought to quantify this by calculating the fractional change in IPSC rise and decay times induced by the temperature rise for each mutant receptor. As shown in Figure 9A, the IPSC rise times for wild type and all mutant GABA_A_Rs were decreased by ~50%. This parameter exhibited no mutation-dependence. The decay time constants for IPSCs mediated by α1β2γ2*^N40S^* and α1β2γ2 GABA_A_Rs both decreased to ~22% of room temperature values (Figure 9B). Notably, the α1β2γ2*^N40S^* GABA_A_R is not associated with FS. On the other hand, α1β2γ2*^R43Q^*, α1β2γ2*^P44S^* and α1β2γ2*^R138G^* GABA_A_Rs exhibited a significantly higher rate of IPSC decay relative to α1β2γ2 GABA_A_Rs as temperature was elevated from 22 to 40°C (Figure 9B). Together, these results suggest that mutations that increase the temperature sensitivity of the IPSC decay rate may be critical for the onset of FS.

### Effects of the K289M Mutation on Intrinsic GABA_A_R Biophysical Properties

**Figure 10:**
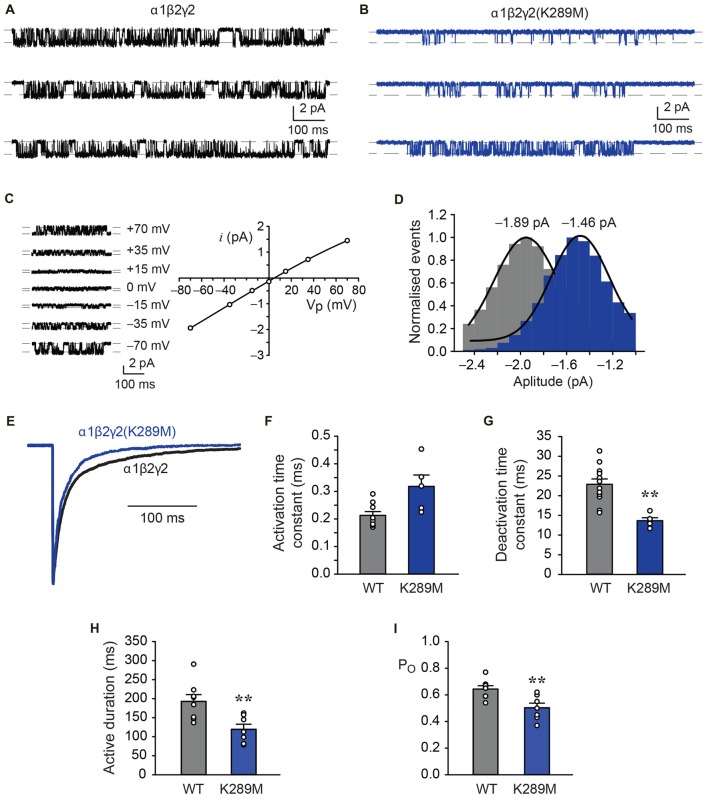
Comparison of the kinetic properties of α1β2γ2 and α1β2γ2*^K289M^* GABA_A_Rs. **(A)** Single channel recordings of wild-type α1β2γ2 receptors in the presence of saturating (3 mM) GABA at a holding potential of −70 mV. **(B)** Single channel recordings of the α1β2γ2*^K289M^* receptors in the presence of saturating (3 mM) GABA at a holding potential of −70 mV. Note the reduced current amplitude and briefer active periods in the mutant receptor. **(C)** Single channel currents recorded at the indicated holding voltages and the group *i*-V for the α1β2γ2 receptors. The reversal potential was 5.0 mV. **(D)** Pooled amplitude histograms for the α1β2γ2 (*n* = 5) and α1β2γ2*^K289M^* (*n* = 7) receptors showing that the K289M mutation reduced the single channel current amplitude (*p* = 0.0002). **(E)** Normalised and overlayed macropatch currents from α1β2γ2 and α1β2γ2*^K289M^* GABA_A_Rs showing that the mutation accelerates the deactivation rate. The currents were elicited by brief exposure (~1 ms) to 3 mM GABA. **(F)** Group data showing that the α1β2γ2 and α1β2γ2*^K289M^* GABA_A_Rs exhibited mean activation times that were not significantly different to each other. **(G)** Group data showing mean deactivation times for α1β2γ2 and α1β2γ2*^K289M^* GABA_A_Rs. The K289M mutation resulted in faster deactivation times compared to wild-type. **(H)** Group data showing the mean active durations of both receptors. The K289M mutation abbreviated the active duration to a significant extent. **(I)** Group data of P_O_ showing that the K289M mutation decreased the time spent in conducting states. **p < 0.005.

The effects of this mutation on functional receptor properties were explored using two methods. First, single receptor currents were recorded in steady-state conditions, and second, fast agonist exchange over macropatches was achieved by exposing the recorded patch to GABA for ~1 ms. In the continuous presence of a saturating (3 mM) GABA concentration, α1β2γ2 GABA_A_Rs activated in discrete periods that were interrupted by brief non-conducting periods of receptor desensitisation (Figure 10A). Similar recordings of α1β2γ2*^K289M^* GABA_A_Rs revealed single channel activity that was of briefer duration and smaller amplitude (Figure 10B). To obtain an estimate of single channel conductance, current-voltage (*i*-V) experiments were carried out for the wild-type receptors over a voltage range from −70 mV to +70 mV as previously described (Keramidas and Harrison, 2010; Dixon et al., 2014). The *i*-V for the α1β2γ2 GABA_A_Rs was near linear and reversed at 5.0 mV (Figure 10C). A net electrical driving force of 79.7 mV (reversal potential of 5.0 mV and liquid junction potential of 4.7 mV) and a mean amplitude of 1.89 ± 0.05 pA (*n* = 5, at −70 mV), yielded a conductance of 23.7 pS. In contrast, the α1β2γ2*^K289M^* GABA_A_Rs had a mean amplitude of 1.46 ± 0.05 pA (*n* = 7, Figure 10D) and a conductance of 18.3 pS. Although relatively small in magnitude, this difference was statistically significant as revealed by an unpaired *t*-test (*p* = 0.0002). The result contrasts with an earlier study that found no significant difference in the single channel conductance (Hales et al., 2006).

Macropatch currents were evinced by applying 3 mM GABA over the recorded patch for ~1 ms (Figure 10E). The activation and deactivation times for α1β2γ2 and α1β2γ2*^K289M^* GABA_A_Rs were determined by fitting exponentials to these phases of the current (Keramidas and Harrison, 2010). This analysis revealed that the activation time for the mutant receptor was unchanged relative to wild-type, with mean values of 0.32 ± 0.04 ms (*n* = 5) and 0.21 ± 0.01 ms (*n* = 9), respectively (Figure 10F). However, the α1β2γ2*^K289M^* GABA_A_Rs deactivated more rapidly than α1β2γ2 GABA_A_Rs, with mean time constants of 13.7 ± 0.7 ms (*n* = 5) and 22.9 ± ms (*n* = 12), respectively (Figures 10E,G). As macropatch deactivation times correspond to single receptor mean activation periods (Scott et al., 2015; Atif et al., 2017; Dixon et al., 2015a), single receptor recordings were also analysed for mean active duration and intra-activation open probability (P_O_). The mean active duration of individual receptors was significantly reduced from 193 ± 18 ms (*n* = 8) for α1β2γ2 GABA_A_Rs to 119 ± 13 ms (*n* = 8, *p* = 0.0067) for α1β2γ2*^K289M^* GABA_A_Rs (Figure 10H). A measurement of the P_O_ also revealed that the mutant receptors had significantly reduced the time spent in conducting configurations within active periods from 0.64 ± 0.02 (*n* = 8) for α1β2γ2 GABA_A_Rs to 0.50 ± 0.03 (*n* = 7, *p* = 0.0052) for α1β2γ2*^K289M^* GABA_A_Rs (Figure 10I). These later results are in general agreement with an earlier analysis of single channel open times (Hales et al., 2006).

The effects of the γ2*^K289M^* mutation have been examined previously in receptors containing α1 and β3 subunits (Bianchi et al., 2002). Here too, the mutation reduced the deactivation times by about 2-fold, but due to the inclusion of the β3 subunit, the deactivation times were ~3-fold slower than those reported here for β2-containing receptors (Bianchi et al., 2002; Chen et al., 2017b). No change in single channel current amplitude was reported for α1β3γ2*^K289M^* GABA_A_Rs (Bianchi et al., 2002), in contrast to the modest, but significant decrease observed here. In summary, the experiments reveal that when the γ2*^K289M^* mutation combines with α1 and β2 subunits, the resultant receptors exhibit a reduced single channel conductance, briefer active periods, a lower P_O_ and faster deactivation times, all of which would contribute to impaired GABAergic inhibition.

## Discussion

In a previous study (Dixon et al., 2014) we reported that with a transfection ratio of 1:1:3 (α1:β2:γ2) approximately 10% of GABA_A_Rs expressed at the cell surface were α1β2 diheteromers. This was determined by analysis of single channel conductances, which are doubled in magnitude when γ2 subunits are incorporated. Here we employed a ratio of 1:1:4 to minimise the occurrence of diheteromeric receptors. On the other hand, it has been shown that overexpression of γ2 subunits may result in an increased occurrence of receptors containing two γ2 subunits (Quirk et al., 1994; Benke et al., 1996; Botzolakis et al., 2016; Baur and Sigel, 2017). Incorporation of a second γ2 subunit (to produce α1γ2α1γ2β2 receptors) has been shown to cause a dramatic slowing in the GABAergic current deactivation rate relative to both the standard diheteromeric and triheteromeric GABA_A_R isoforms (i.e., α1β2α1β2β2 or α1β2α1γ2β2 receptors; Botzolakis et al., 2016). Because we observed an acceleration in the deactivation rate when we overexpressed γ2 subunits (Figure 2), we consider it unlikely that the majority of our triheteromeric receptors incorporated two γ2 subunits. We thus consider that our transfection ratio of 1:1:4 resulted predominantly in receptors comprising two α1, two β2 and one γ2 subunit.

The γ2 subunit is important for efficient trafficking of GABA_A_Rs to the cell membrane (Nakamura et al., 2015; Vien et al., 2016) and for GABA_A_R clustering at synapses (Essrich et al., 1998; Alldred et al., 2005). Hereditary epilepsy mutations to γ2 impair channel function by various means including ER retention of misfolded subunits, mutant subunit aggregation, dominant negative suppression of partner subunits, impaired channel gating and aberrant synaptic targeting (Kang and Macdonald, 2016). In the absence of developmental compensations, these effects would lead to a loss of inhibitory tone and thus to disinhibition of neuronal network electrical activity. Although epilepsy-causing γ2 subunit nonsense, frameshift, splice-site and deletion mutations are all known (Hirose, 2014; Kang and Macdonald, 2016), the present study focused on missense mutations because these are likely to form misfolded but full length proteins that have the potential to be corrected by proteostatic enhancers such as SAHA. We also investigated the well-characterised missense gating mutation, K289M, as a control.

It is difficult to study defined GABA_A_R isoforms in native neuronal synapses due: (1) to the multitude of other isoforms present; and (2) the difficulty in pharmacologically or genetically isolating the receptor isoform of interest. We investigated the effects of mutations in artificial synapses because this is the only known way of ascertaining that IPSCs are mediated solely by the receptors formed from the recombinantly expressed subunits. A potential limitation is that they may not precisely replicate neuronal GABAergic synapses given that HEK293 cells do not express all necessary postsynaptic clustering proteins at appropriate levels. However, we have previously shown that the rise and decay times of IPSCs mediated by α1β2γ2 GABA_A_Rs in artificial synapses are identical to those of native synapses where it has been possible to ascertain that the α1β2γ2 GABA_A_R is the major isoform present (Nusser et al., 1997; Okada et al., 2000; Eyre et al., 2012; Dixon et al., 2014). In addition, electron microscopy reconstructions have shown that GABAergic artificial synapses have ultrastructures similar to those of neurons (Fuchs et al., 2013).

One confound with GABAergic artificial synapses is that α1β2 diheteromeric GABA_A_Rs mediate large slow IPSCs. This does not appear to happen in native neuronal synapses. For example, in γ2^−/–^ mice, the frequency of GABAergic IPSCs is reduced by ~80% despite the extrasynaptic expression of diheteromeric GABA_A_Rs remaining high (Gunther et al., 1995; Essrich et al., 1998). Thus, in contrast to artificial synapses, knock down of γ2 subunits in neurons does not result in large slow IPSCs mediated by α1β2 GABA_A_Rs. Thus, a limitation of the present study is that, due to contamination by α1β2-mediated currents, we cannot make any inferences about the properties of IPSCs mediated by triheteromeric GABA_A_Rs that incorporate epilepsy mutant γ2 subunits with reduced surface expression. We can, however, draw inferences about the effect of SAHA on mutant γ2 subunit surface expression levels and the effects of enhanced mutant γ2 incorporation on IPSC kinetics.

Consistent with previous findings, our measurements showed that all mutations apart from K289M were poorly expressed in the absence of SAHA. That is, their IPSC rise and decay times tended towards those of α1β2 rather than α1β2γ2 GABA_A_Rs. Judging by the magnitude of the shift towards α1β2 properties, we conclude that R43Q exerted the most deleterious effect on expression, with N40S, P44S and R138G also showing significant impairment (Figure 1). These findings were supported by immunohistochemistry (Figure 2), and correspond reasonably well with previous studies that showed largest (up to 90%) surface expression reductions for R43Q and P44S (Huang et al., 2014), a 60% reduction for R138G (Todd et al., 2014) and a small (12%) reduction for N40S (Huang et al., 2014).

SAHA has previously been shown to have a minimal effect on the expression of α1β2γ2 GABA_A_Rs (Di et al., 2013) and here we observed no significant effect of SAHA on α1β2 or α1β2γ2*^K289M^* GABA_A_Rs (Figures 3, 8). We thus infer that SAHA acted specifically on ER-retained misfolded γ2 subunits. In the case of GABA_A_Rs formed following transfection with α1, β2 and N40S, P44S or R138G mutant γ2 subunits, SAHA completely reverted the rise and decay time constants to wild type triheteromeric receptor values (Figures 4, 6, 7). This suggests either a dramatic (perhaps complete) upregulation of mutant γ2 surface expression or a mixture of moderate upregulation coupled with an accelerating effect of the mutation on the intrinsic activation and deactivation rates. In the case of the α1β2γ2*^R43Q^* GABA_A_R, the incomplete reversion of the decay time constant (Figure 5D) implies a partial recovery of expression or a slowing in the deactivation rate caused by the R43Q mutation.

Our results for the α1β2γ2*^K289M^* GABA_A_R generally concur well with previous studies (Bianchi et al., 2002; Hales et al., 2006; Eugene et al., 2007; Bouthour et al., 2012). In artificial synapses we found the mutation significantly accelerated the IPSC decay rate (Figure 8D) whereas in single channel and fast application experiments we showed that this was due to briefer active periods, a lower P_O_ and faster deactivation times (Figure 10). The only result that contrasted with previous studies (Bianchi et al., 2002; Hales et al., 2006) was a small but significant reduction in unitary conductance. It is not surprising that the deletion of a positive charge at a site close to the pore vestibule would reduce the conductance of an anion-selective channel (Imoto et al., 1988; Scott et al., 2015).

Our results may provide some insight into the mechanisms of FS. The SAHA-treated α1β2γ2*^N40S^* and α1β2γ2 GABA_A_Rs exhibited indistinguishable IPSC rise and decay rates at 22 and 40°C. This result fits well with the lack of association between N40S and FS. In contrast, the α1β2γ2*^R43Q^*, α1β2γ2*^P44S^* and α1β2γ2*^R138G^* GABA_A_Rs mediated IPSCs that all decayed at significantly greater rates than wild type receptors as temperature was increased from 22 to 40°C. This may help explain why epileptic seizures are triggered by fever in patients with these mutations. In contrast, the α1β2γ2*^K289M^* GABA_A_R, which is also associated with FS, exhibited an IPSC decay rate that reduced in parallel with the wild type receptor over the same temperature range. However, it may be relevant that K289M-containing receptors exhibit faster decay rates at both 22 and 40°C. Our results suggest that IPSCs that decay faster than wild type at temperatures above 37°C could trigger FS. However, other factors may also be important in this respect. For example, it has previously been shown at high temperatures the K289M mutation reduced the frequency of mIPSCs in neurons and decreased synaptic clustering due to faster diffusion of individual GABA_A_Rs (Bouthour et al., 2012). These effects are yet to be tested for GABA_A_Rs containing other mutant subunits associated with FS.

In conclusion, we have shown that SAHA enhances the surface expression of mutant triheteromeric α1β2γ2*^N40S^*, α1β2γ2*^R43Q^*, α1β2γ2*^P44S^* and α1β2γ2*^R138G^* GABA_A_Rs in artificial synapses. Indeed, the surface expression of α1β2γ2*^N40S^*, α1β2γ2*^P44S^* and α1β2γ2*^R138G^* GABA_A_Rs is enhanced to the point where they mediate IPSCs with identical rise and decay times to those mediated by wild type α1β2γ2 GABA_A_Rs. Given that SAHA readily crosses the blood-brain barrier and is approved by therapeutic regulatory agencies worldwide for human internal use, it seems reasonable to propose that it may be worth investigating as a treatment for epilepsies caused by these mutations. Although SAHA has already been proposed as a candidate therapeutic for the α1^A295D^ subunit epilepsy mutation (Di et al., 2013; Chen et al., 2017a), the present study extends its potential utility to a new subunit and four new mutations. This prompts us to speculate that proteostasis-enhancing drugs may be worth considering for any GABA_A_R epilepsy mutation associated with protein misfolding and ER retention.

## Author Contributions

ND, CLD, AK and JWL conceived the project and designed the experiments. CLD performed mutagenesis. ND performed and analyzed heterosynapse experiments. ND performed and analyzed microscopy experiments. AK performed and analyzed single channel and rapid application experiments. ND, AK and JWL interpreted data, wrote and edited the manuscript.

## Conflict of Interest Statement

The authors declare that the research was conducted in the absence of any commercial or financial relationships that could be construed as a potential conflict of interest.
